# Supplementary material for: Bayesian refinement of protein structures and ensembles against SAXS data using molecular dynamics
Source: PLoS Comput Biol. 2017 Oct 18;13(10):e1005800. doi: 10.1371/journal.pcbi.1005800 (PMC5662244; doi:10.1371/journal.pcbi.1005800)
Supplement: S1 Archive — (BZ2) [file pcbi.1005800.s011.tar.bz2 › gromacs_4.62_bayesian_saxs_refi/share/html/online/g_x2top.html]

g\_x2top


| g\_x2top Main Table of Contents |

**VERSION 4.6  
Sat 19 Jan 2013**

---

### Description

g\_x2top generates a primitive topology from a coordinate file.
The program assumes all hydrogens are present when defining
the hybridization from the atom name and the number of bonds.
The program can also make an .rtp entry, which you can then add
to the .rtp database.

When -param is set, equilibrium distances and angles
and force constants will be printed in the topology for all
interactions. The equilibrium distances and angles are taken
from the input coordinates, the force constant are set with
command line options.
The force fields somewhat supported currently are:

G53a5 GROMOS96 53a5 Forcefield (official distribution)

oplsaa OPLS-AA/L all-atom force field (2001 aminoacid dihedrals)

The corresponding data files can be found in the library directory
with name atomname2type.n2t. Check Chapter 5 of the manual for more
information about file formats. By default, the force field selection
is interactive, but you can use the -ff option to specify
one of the short names above on the command line instead. In that
case g\_x2top just looks for the corresponding file.

### Files

| option | filename | type | description |
| --- | --- | --- | --- |
| **-f** | conf.gro | Input | Structure file: gro g96 pdb tpr etc. |
| **-o** | out.top | Output, Opt. | Topology file |
| **-r** | out.rtp | Output, Opt. | Residue Type file used by pdb2gmx |

### Other options

| option | type | default | description |
| --- | --- | --- | --- |
| **-[no]h** | bool | no | Print help info and quit || **-[no]version** | bool | no | Print version info and quit || **-nice** | int | 0 | Set the nicelevel || **-ff** | string | oplsaa | Force field for your simulation. Type "select" for interactive selection. || **-[no]v** | bool | no | Generate verbose output in the top file. || **-nexcl** | int | 3 | Number of exclusions || **-[no]H14** | bool | yes | Use 3rd neighbour interactions for hydrogen atoms || **-[no]alldih** | bool | no | Generate all proper dihedrals || **-[no]remdih** | bool | no | Remove dihedrals on the same bond as an improper || **-[no]pairs** | bool | yes | Output 1-4 interactions (pairs) in topology file || **-name** | string | ICE | Name of your molecule || **-[no]pbc** | bool | yes | Use periodic boundary conditions. || **-[no]pdbq** | bool | no | Use the B-factor supplied in a .pdb file for the atomic charges || **-[no]param** | bool | yes | Print parameters in the output || **-[no]round** | bool | yes | Round off measured values || **-kb** | real | 400000 | Bonded force constant (kJ/mol/nm^2) || **-kt** | real | 400 | Angle force constant (kJ/mol/rad^2) || **-kp** | real | 5 | Dihedral angle force constant (kJ/mol/rad^2) |

### Known problems

- The atom type selection is primitive. Virtually no chemical knowledge is used- Periodic boundary conditions screw up the bonding- No improper dihedrals are generated- The atoms to atomtype translation table is incomplete (atomname2type.n2t file in the data directory). Please extend it and send the results back to the GROMACS crew.

---

http://www.gromacs.org  
gromacs@gromacs.org
